# Supplementary material for: Estimating malaria antigen dynamics and the time to negativity of next-generation malaria rapid diagnostic tests
Source: Malar J. 2025 Apr 4;24:109. doi: 10.1186/s12936-025-05350-5 (PMC11969773; doi:10.1186/s12936-025-05350-5)
Supplement: Supplementary file 1 — Additional file 1 [file 12936_2025_5350_MOESM1_ESM.docx]

**Supplementary Materials**


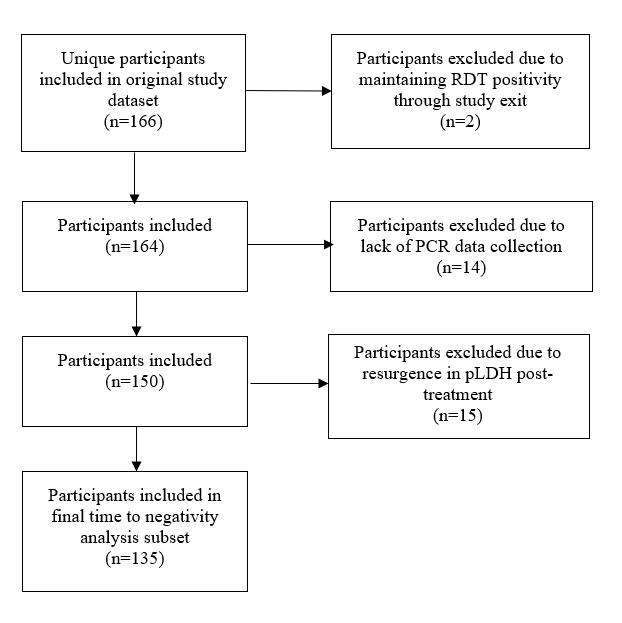


**Supplementary Fig. 1** PRISMA-flow style diagram depicting exclusion and inclusion decision flow for participants from the initial study by Ntuku et al. [4]. Numbers differ from final inclusion in Ntuku et al. due to separate requirements for individuals to have begun submitting samples on Day 1 of enrollment in study.


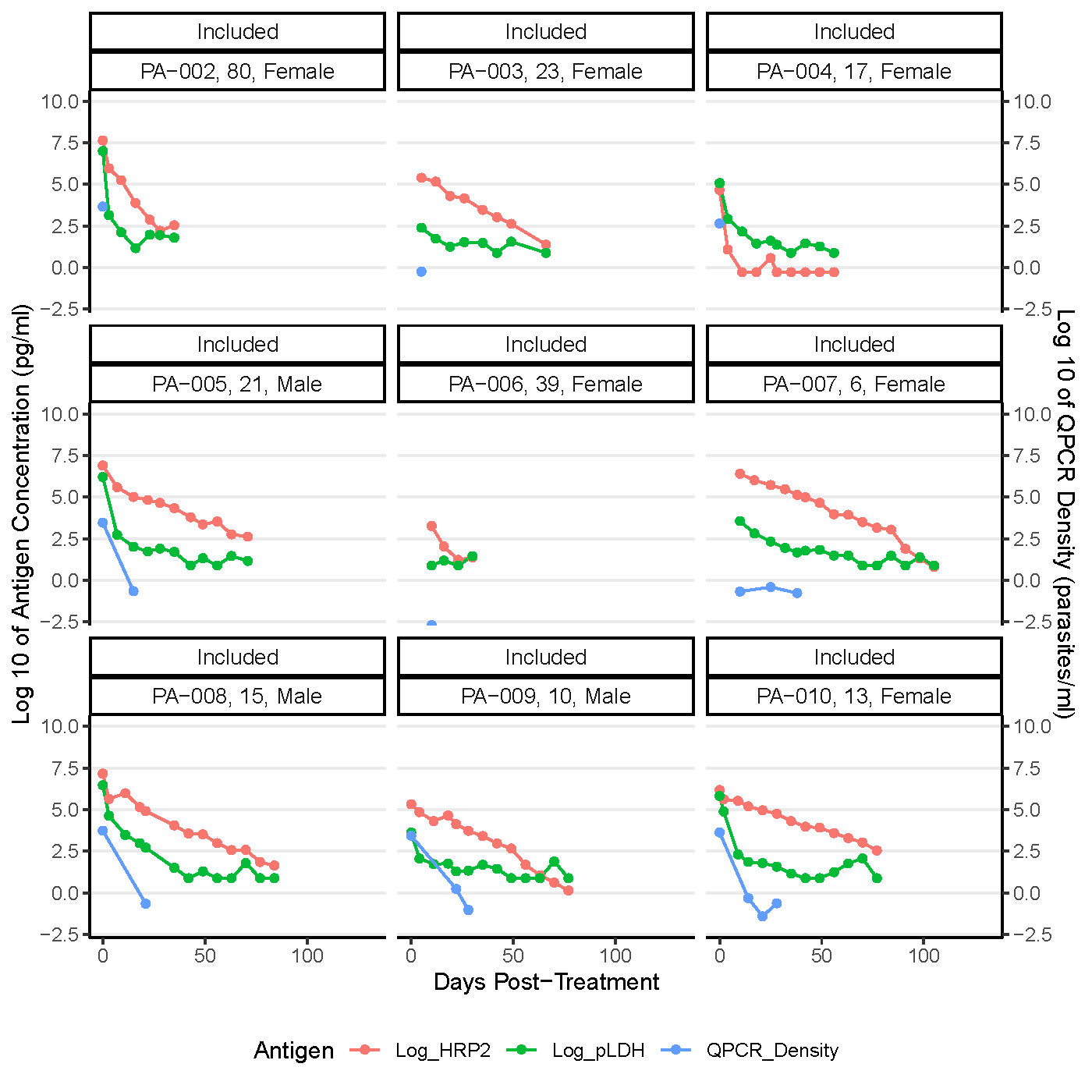

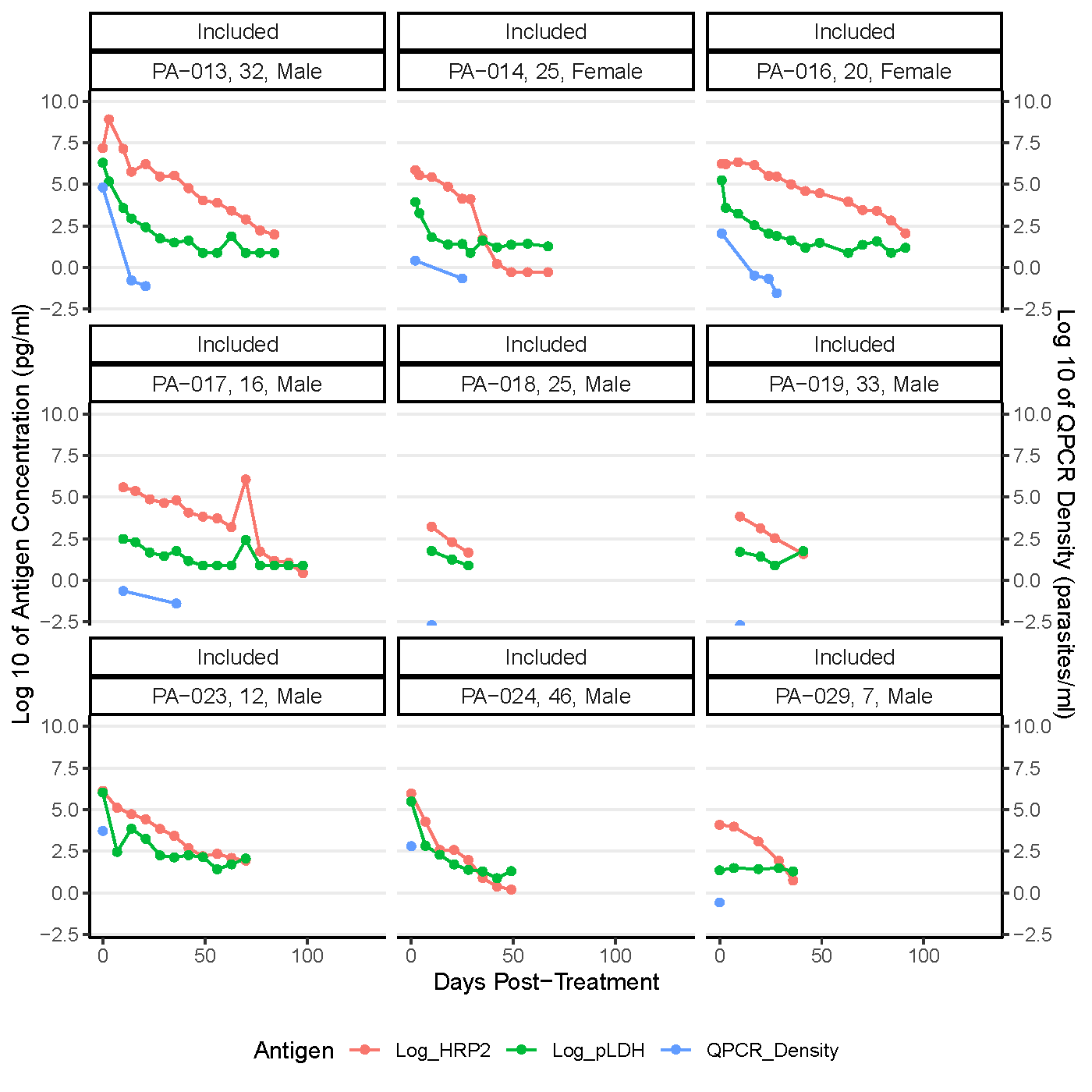

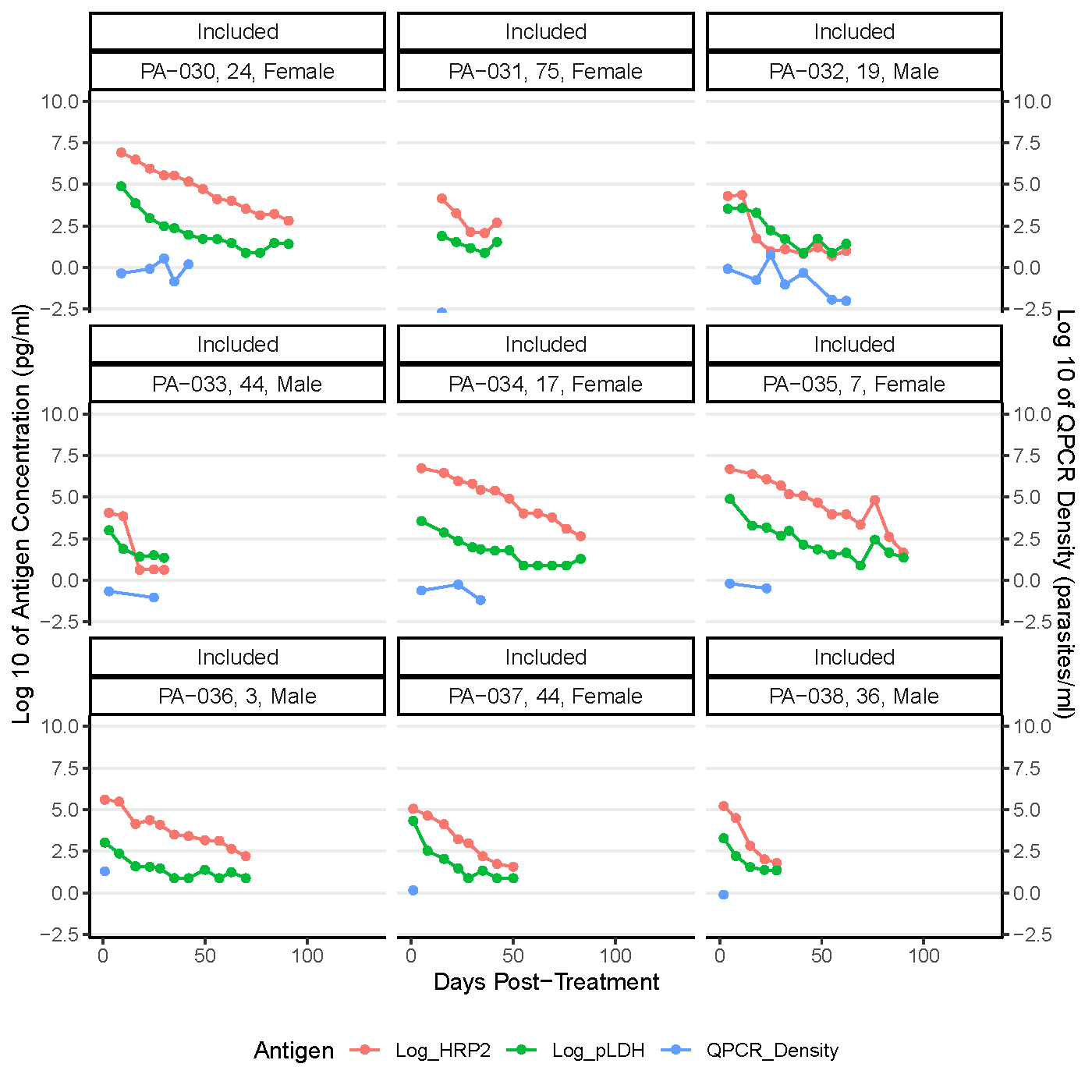

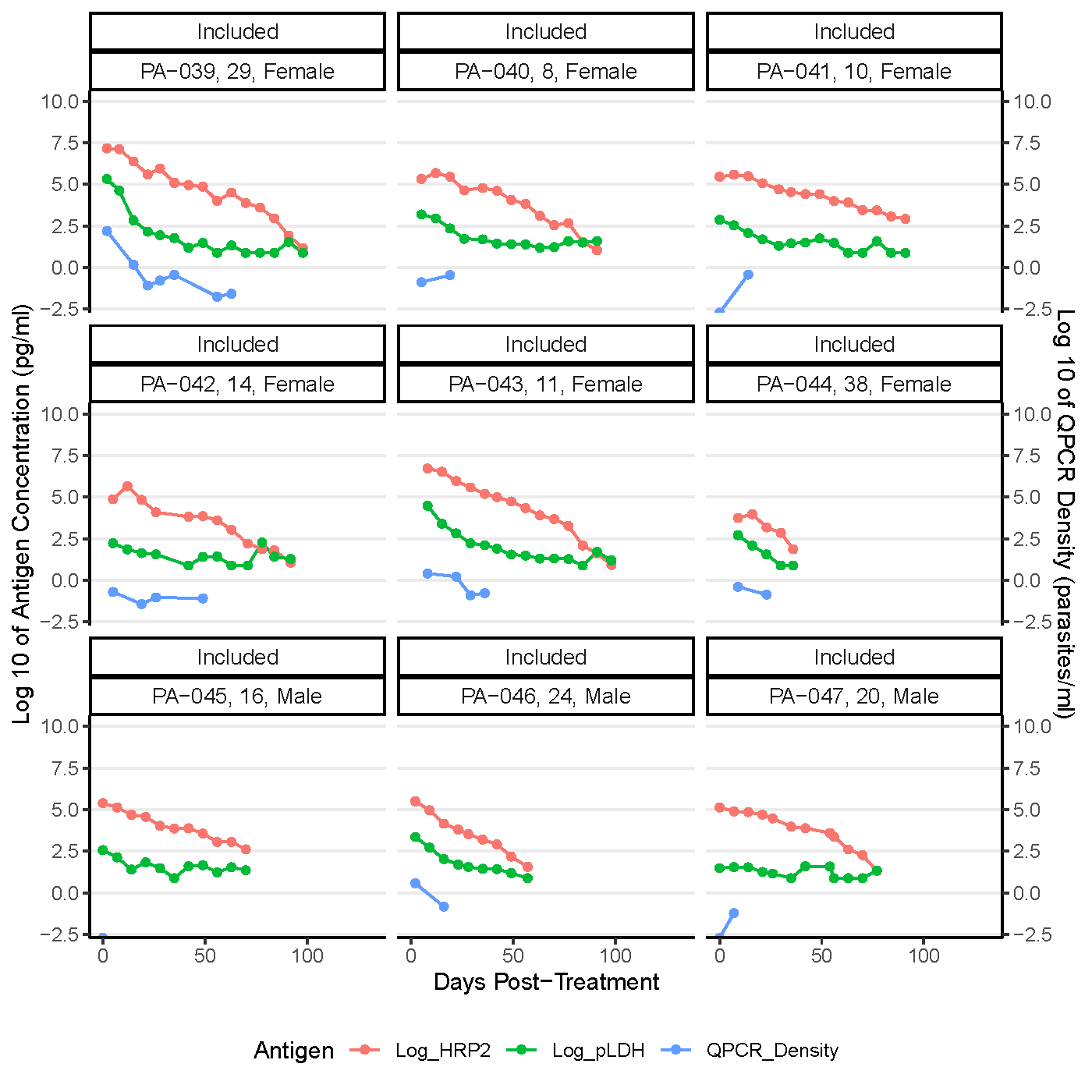

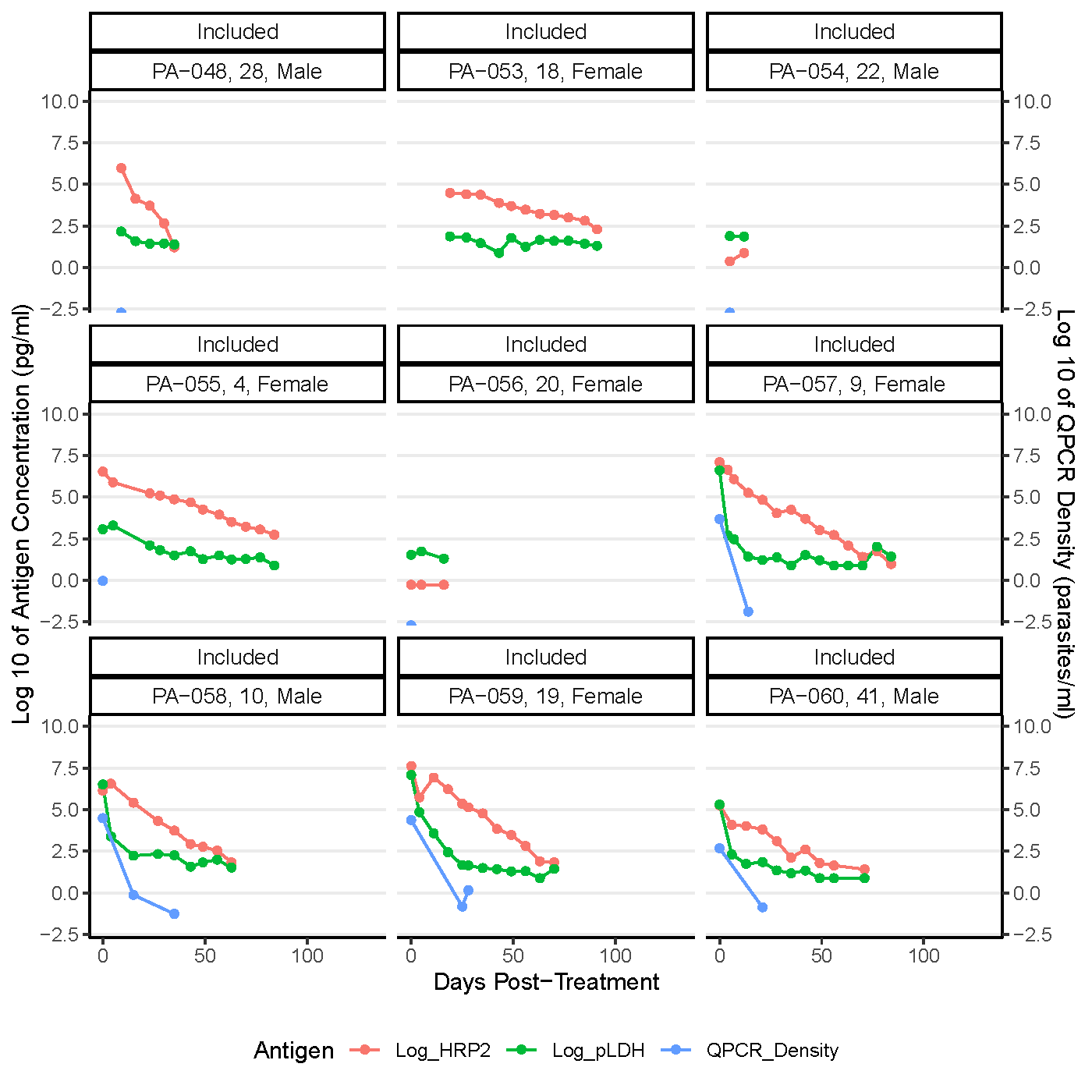

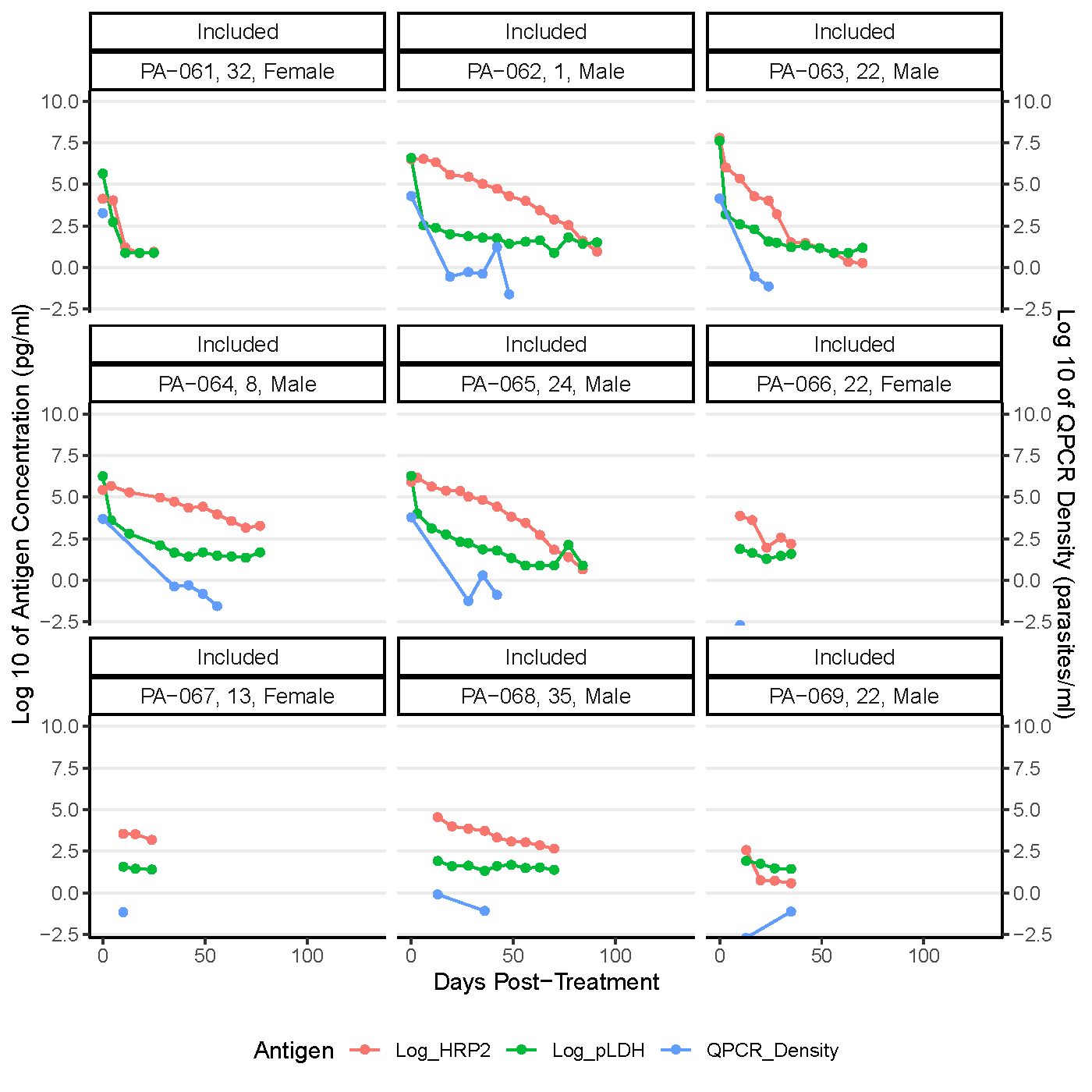

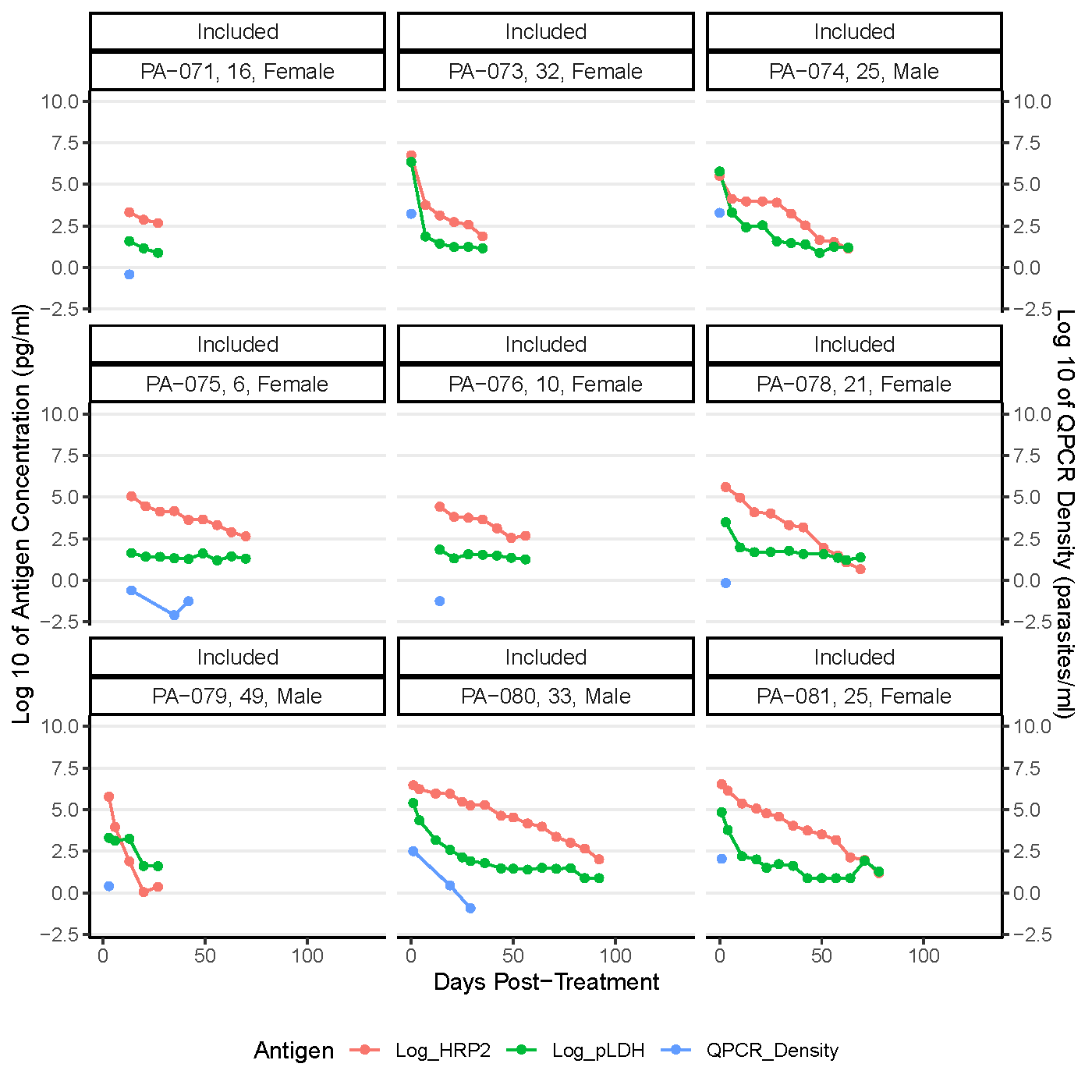

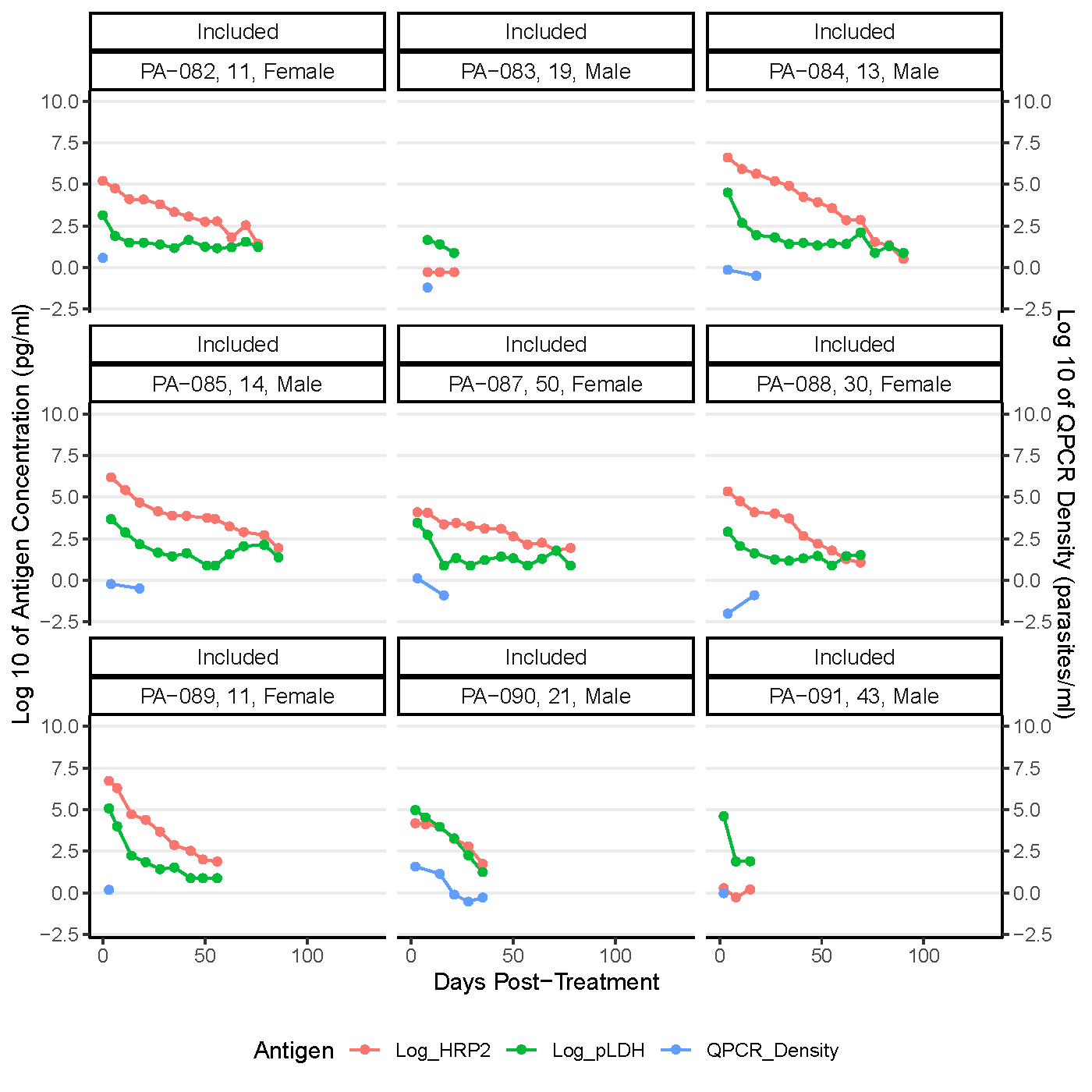

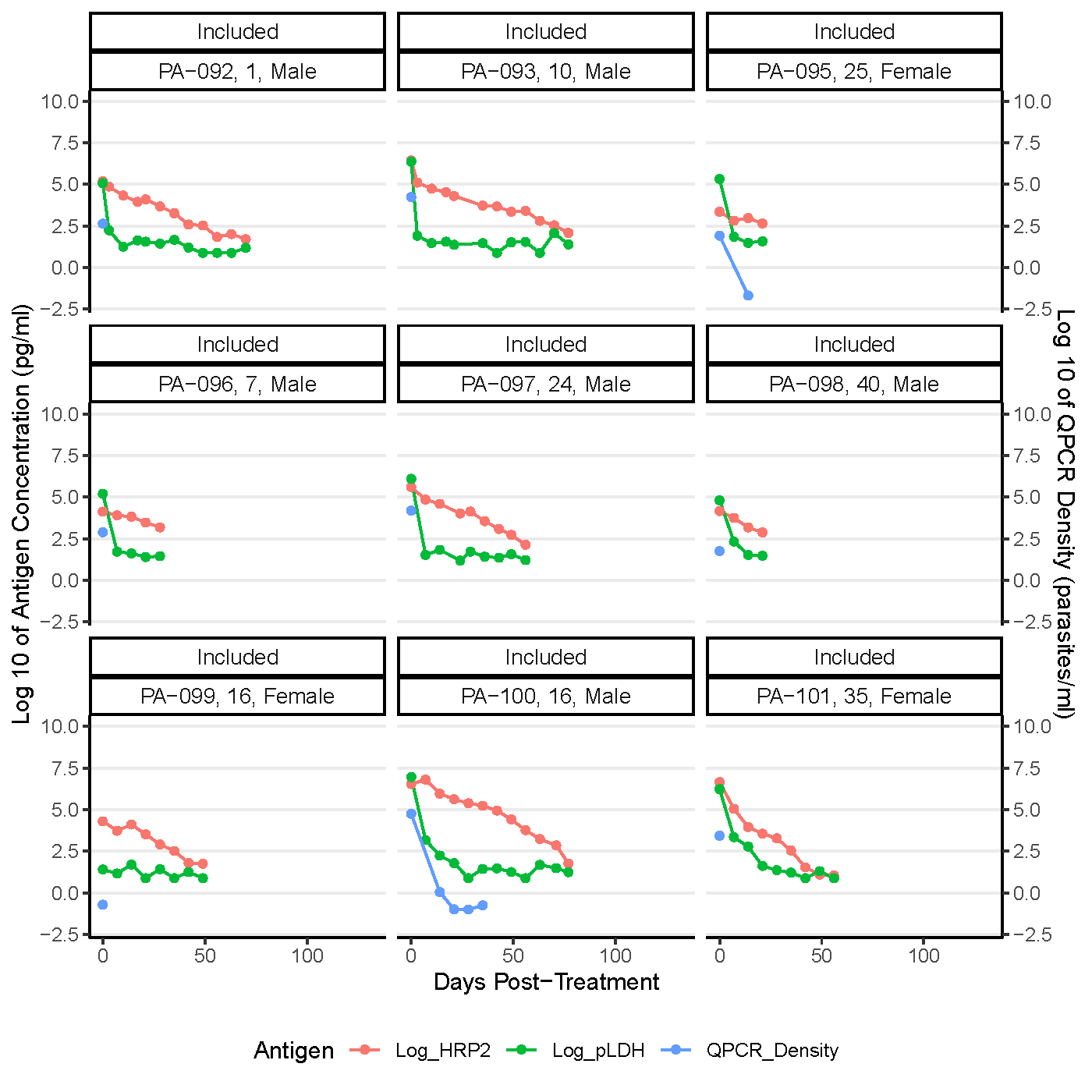

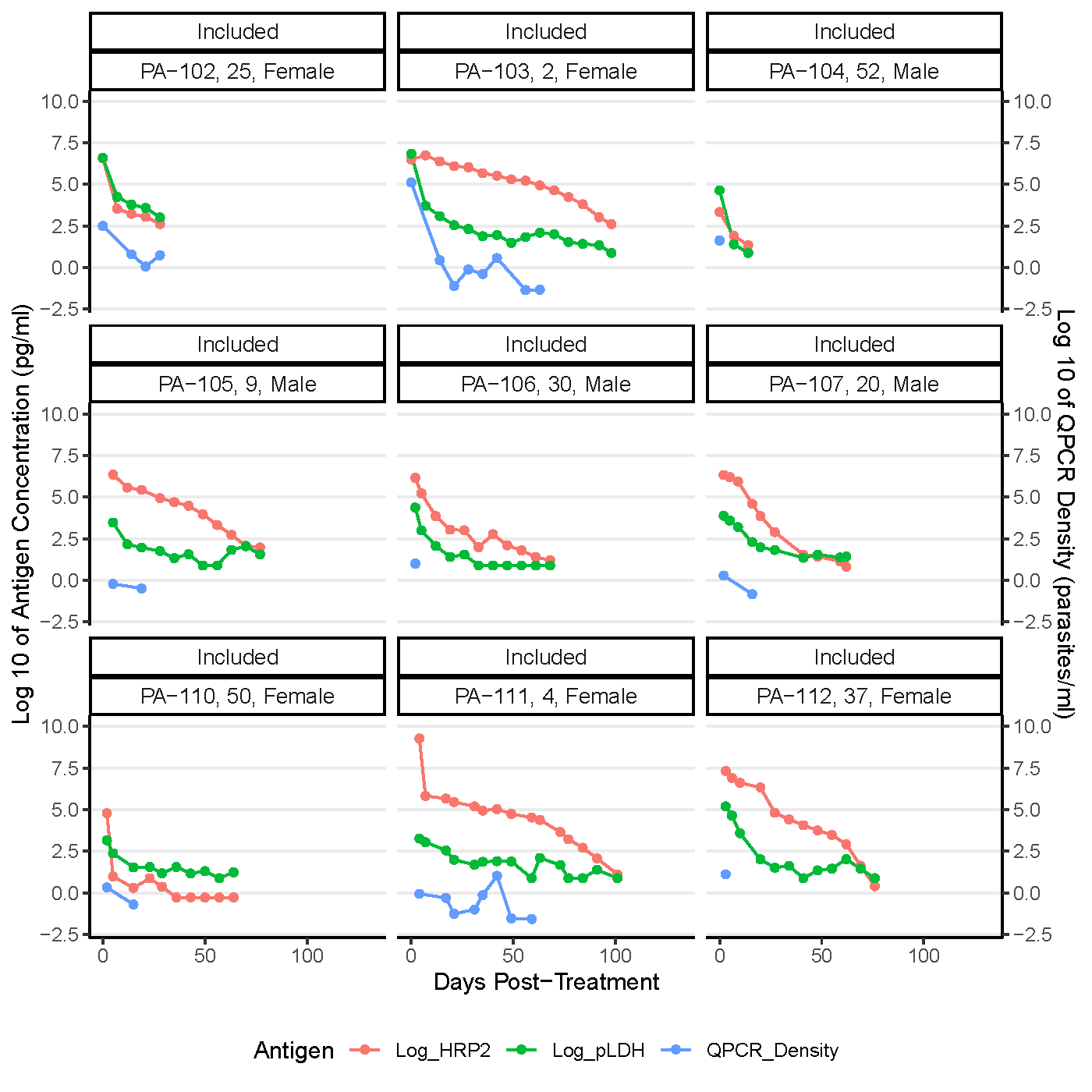

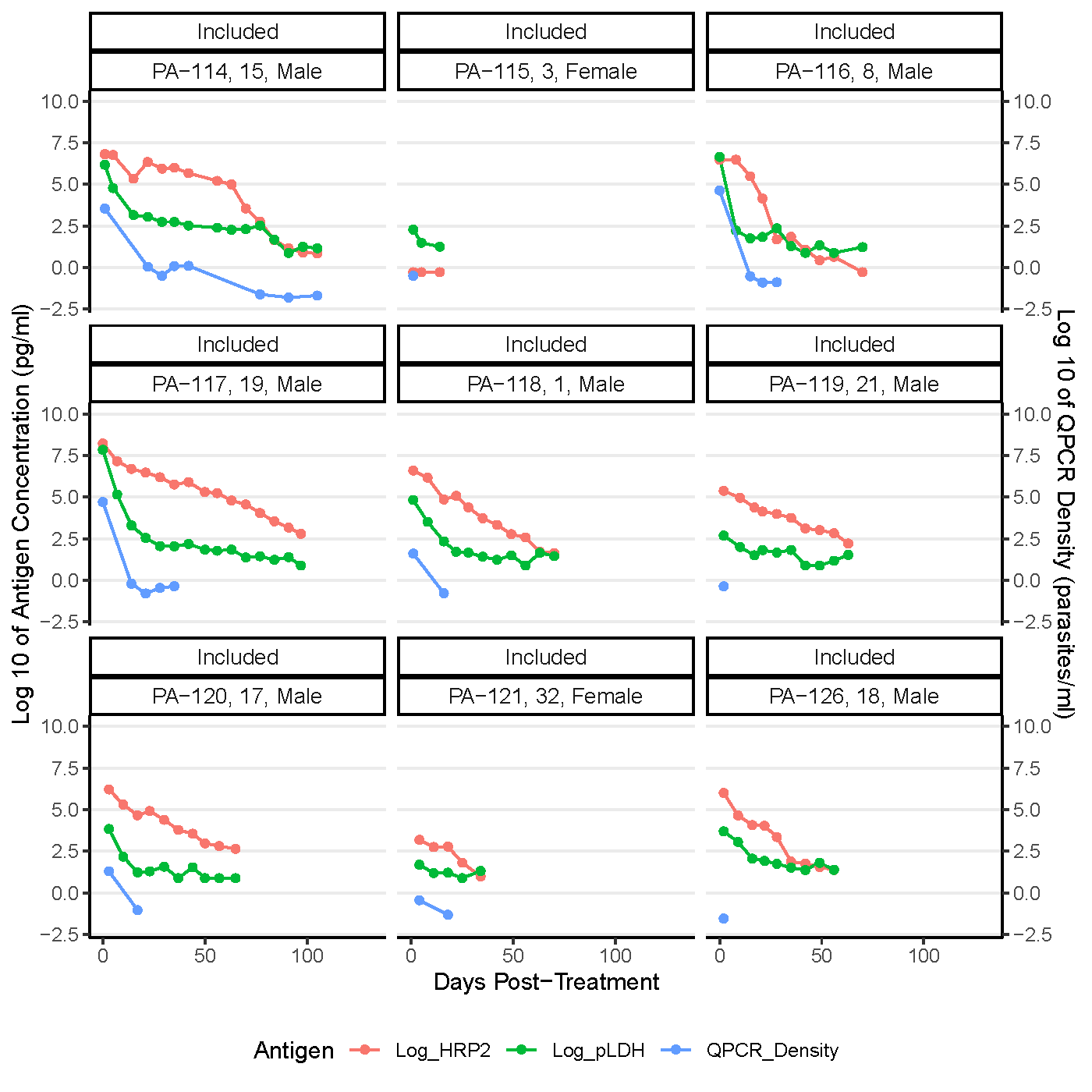

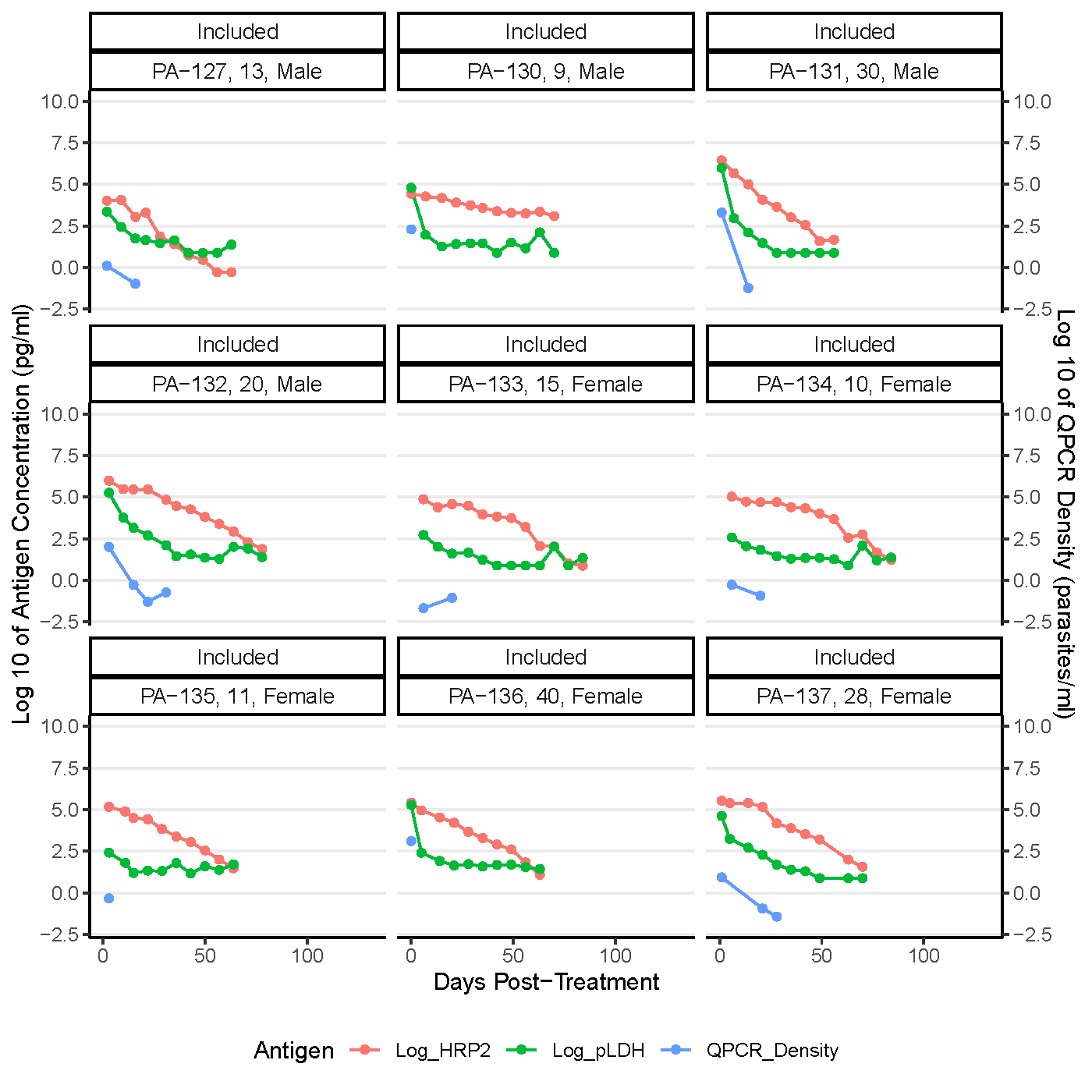

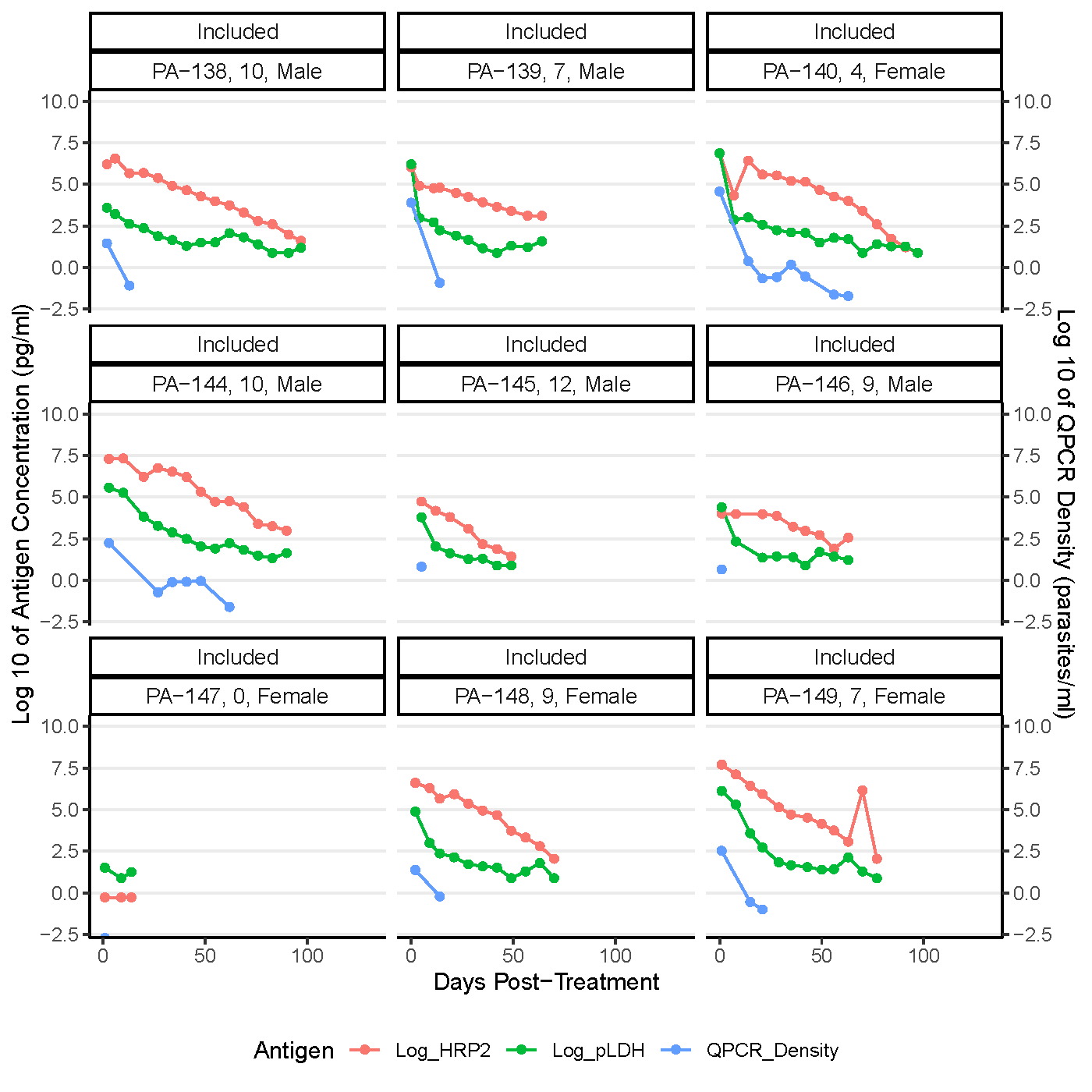

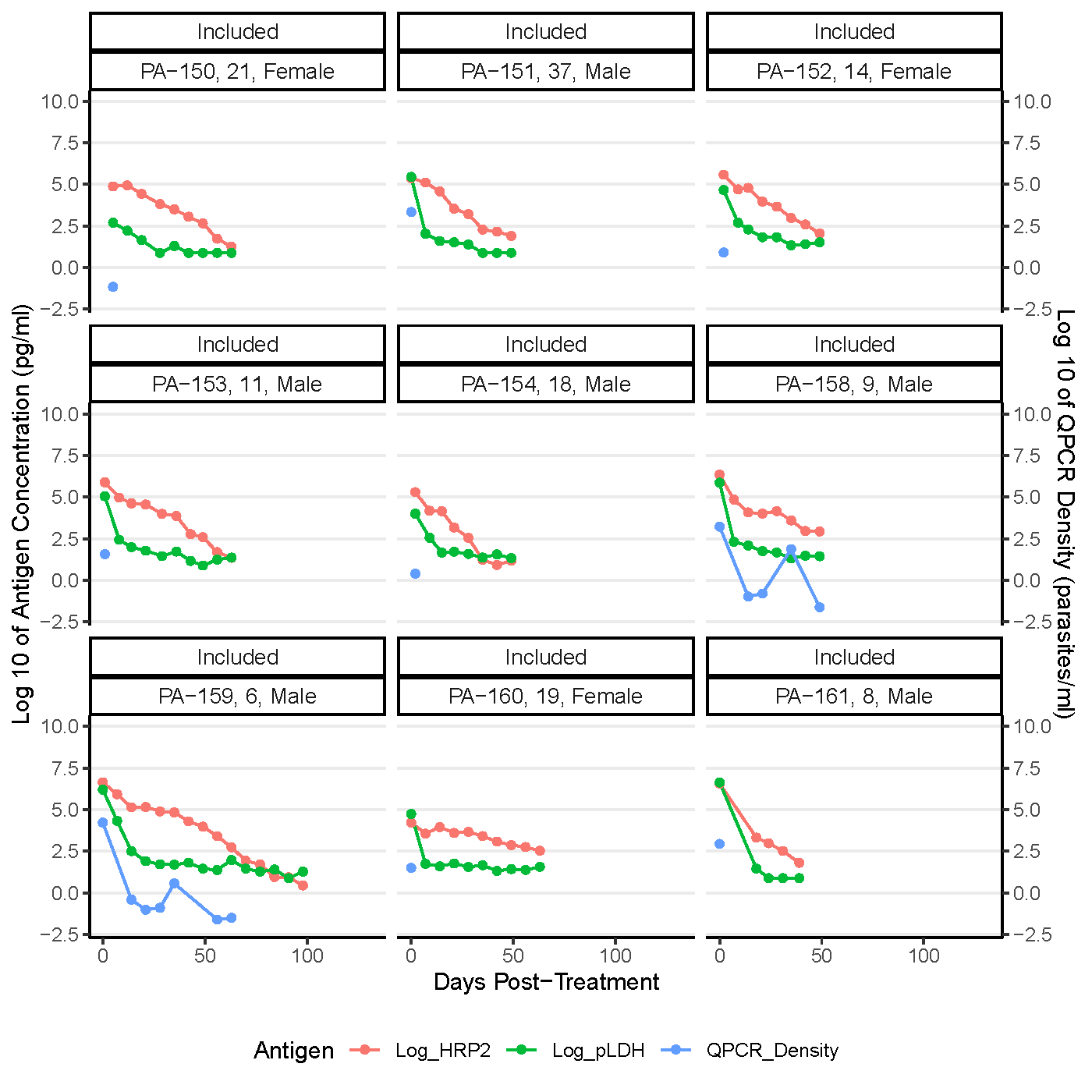

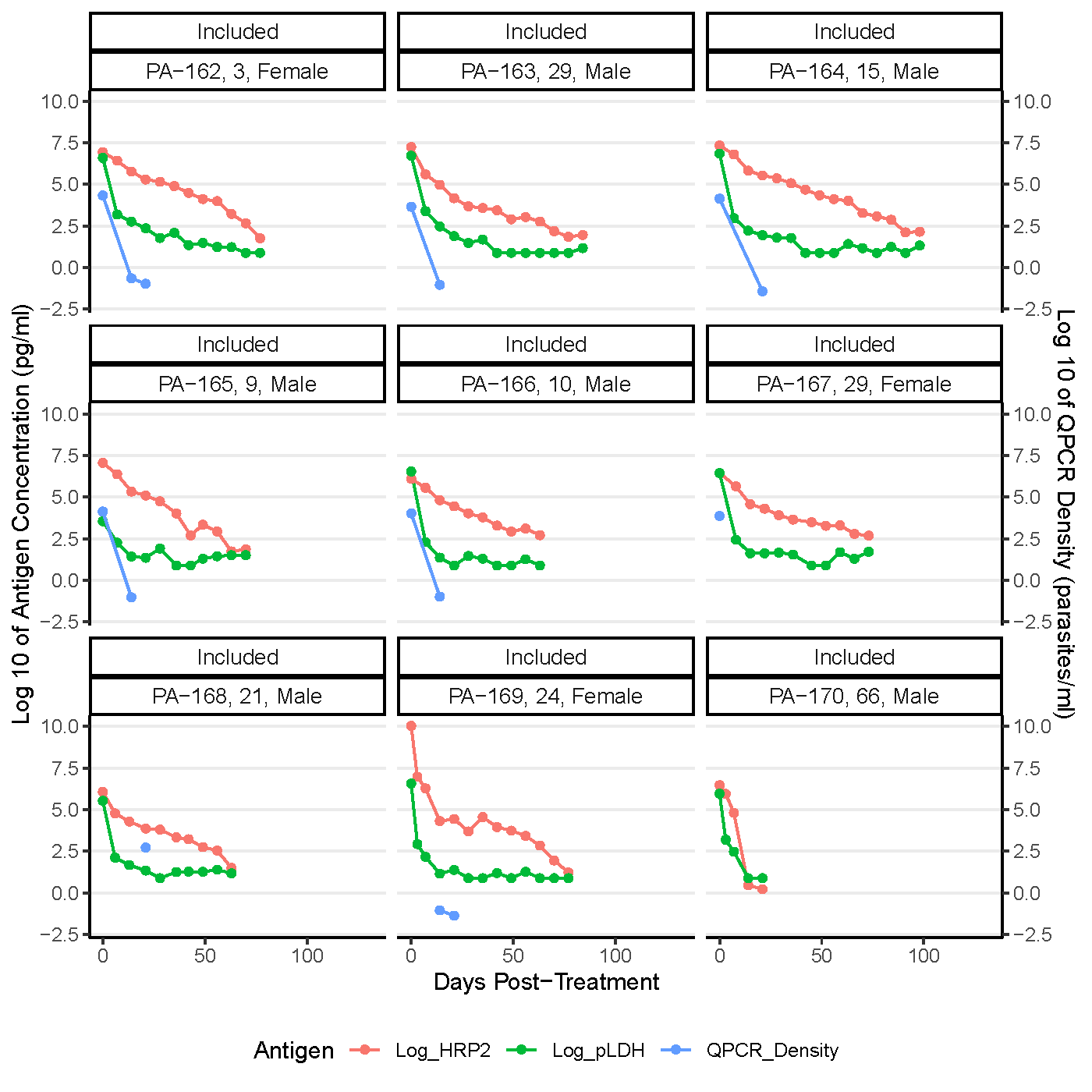

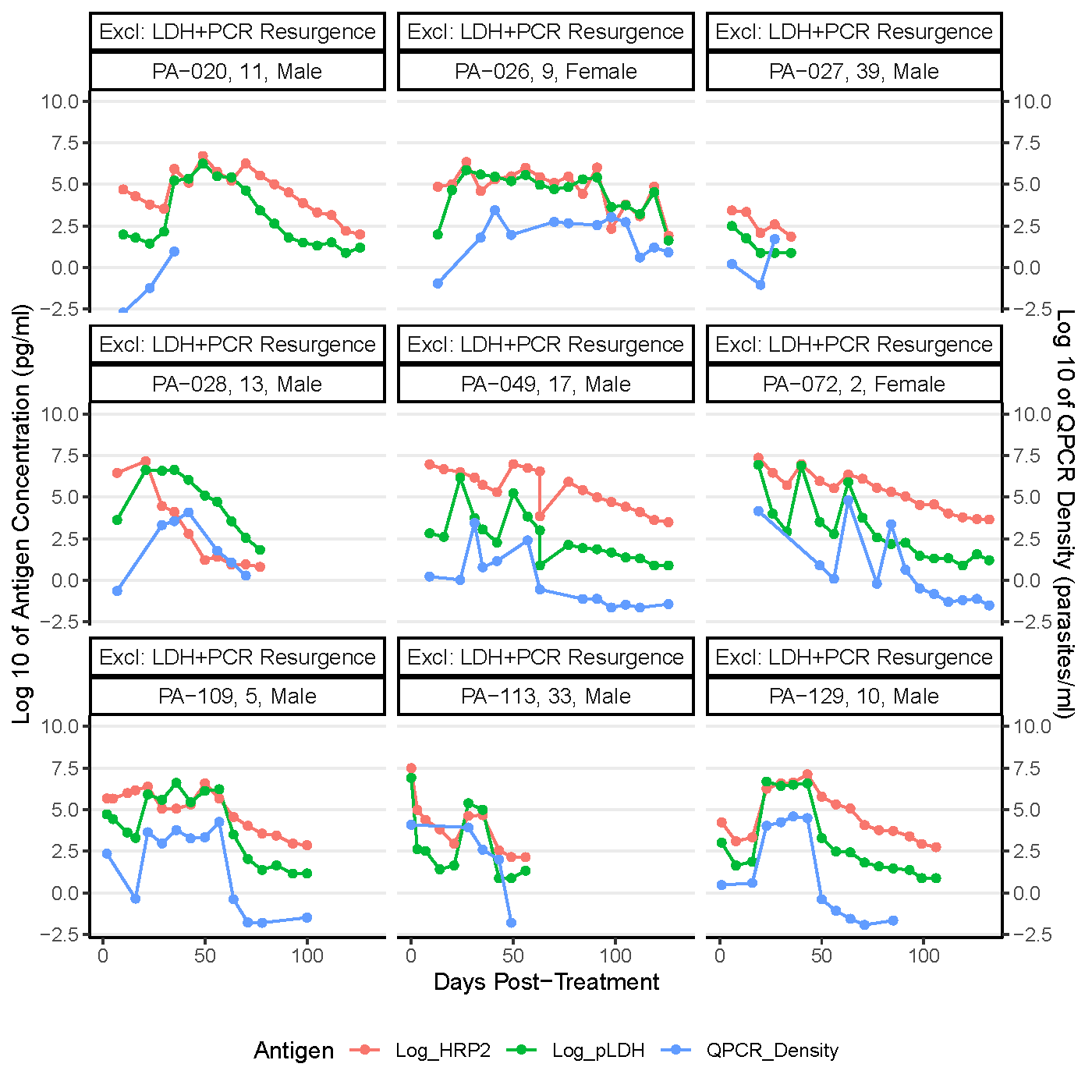

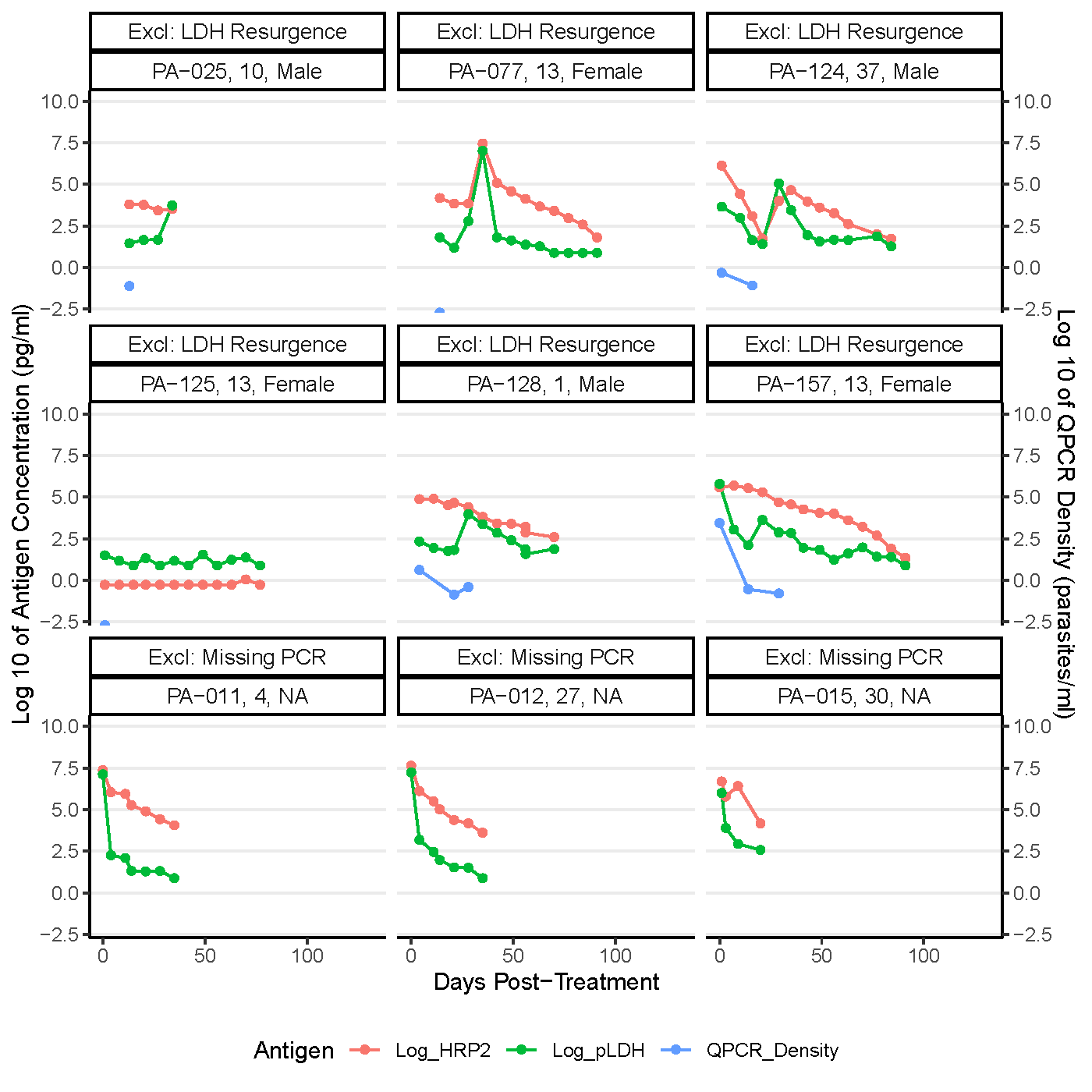

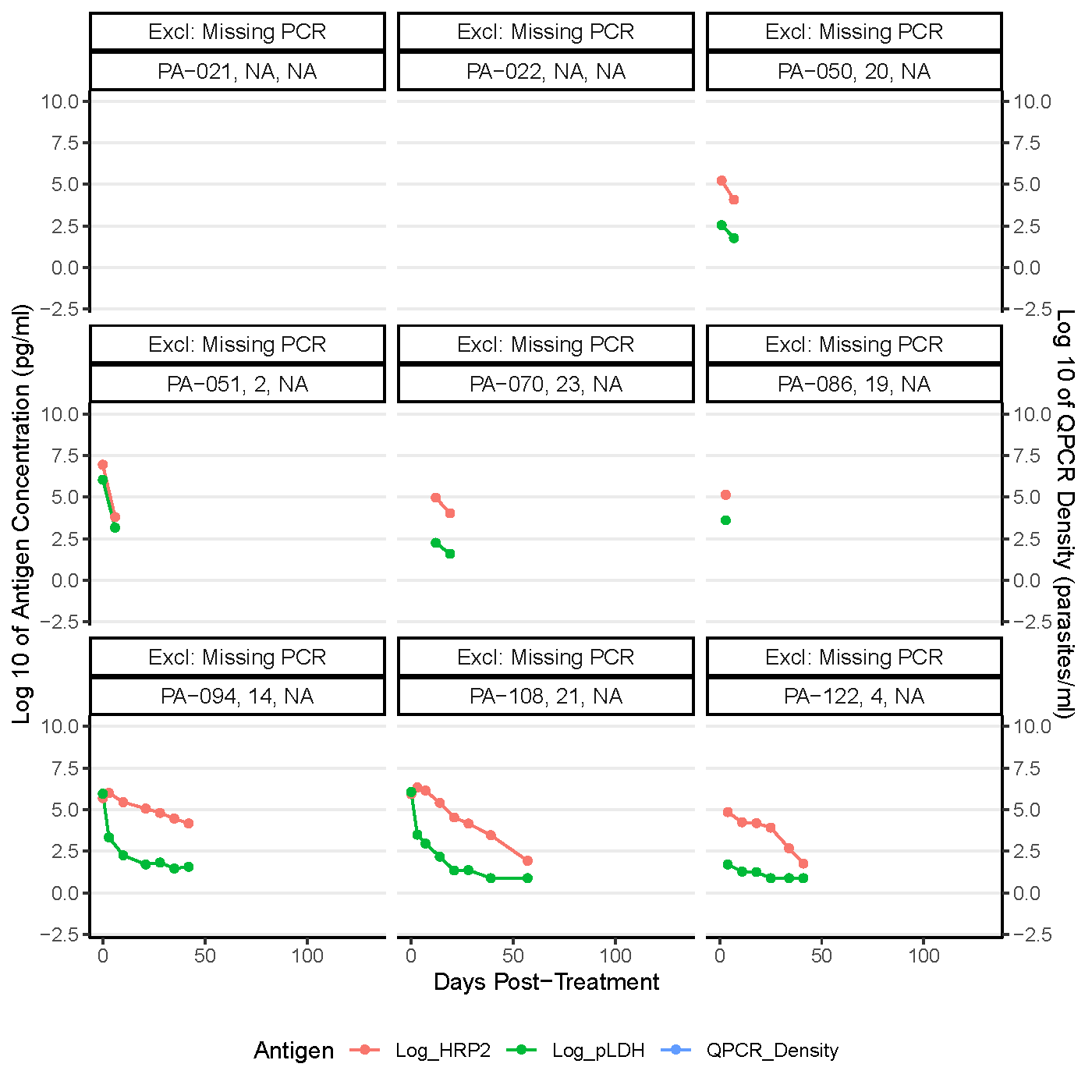

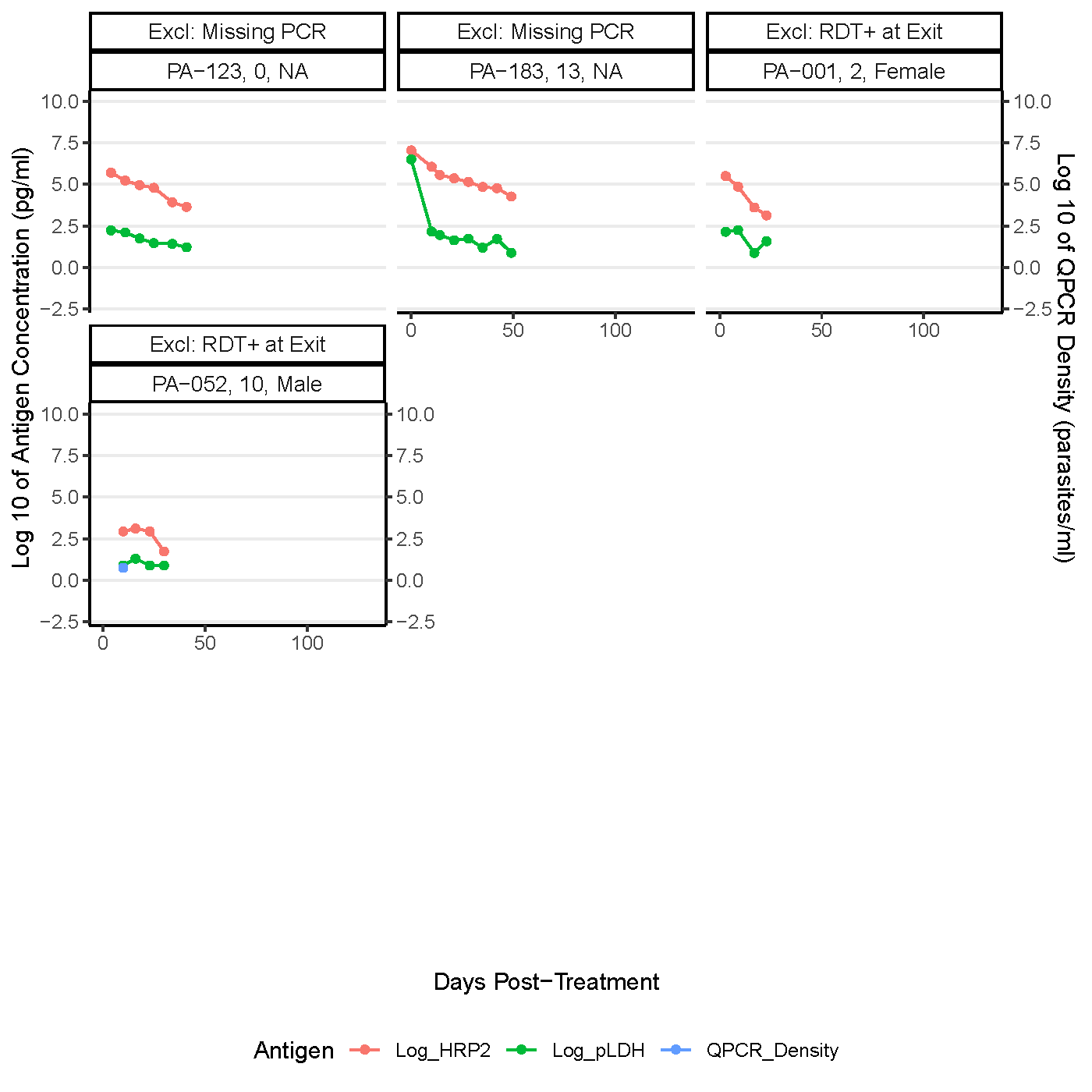


**Supplementary Fig. 2** Individual participant antigen dynamic profiles showing the log-transformed HRP2 and PfLDH concentrations for each participant over time, as well as QPCR density where available. Each participant has their ID number, age, sex, and exclusion criteria labeled.


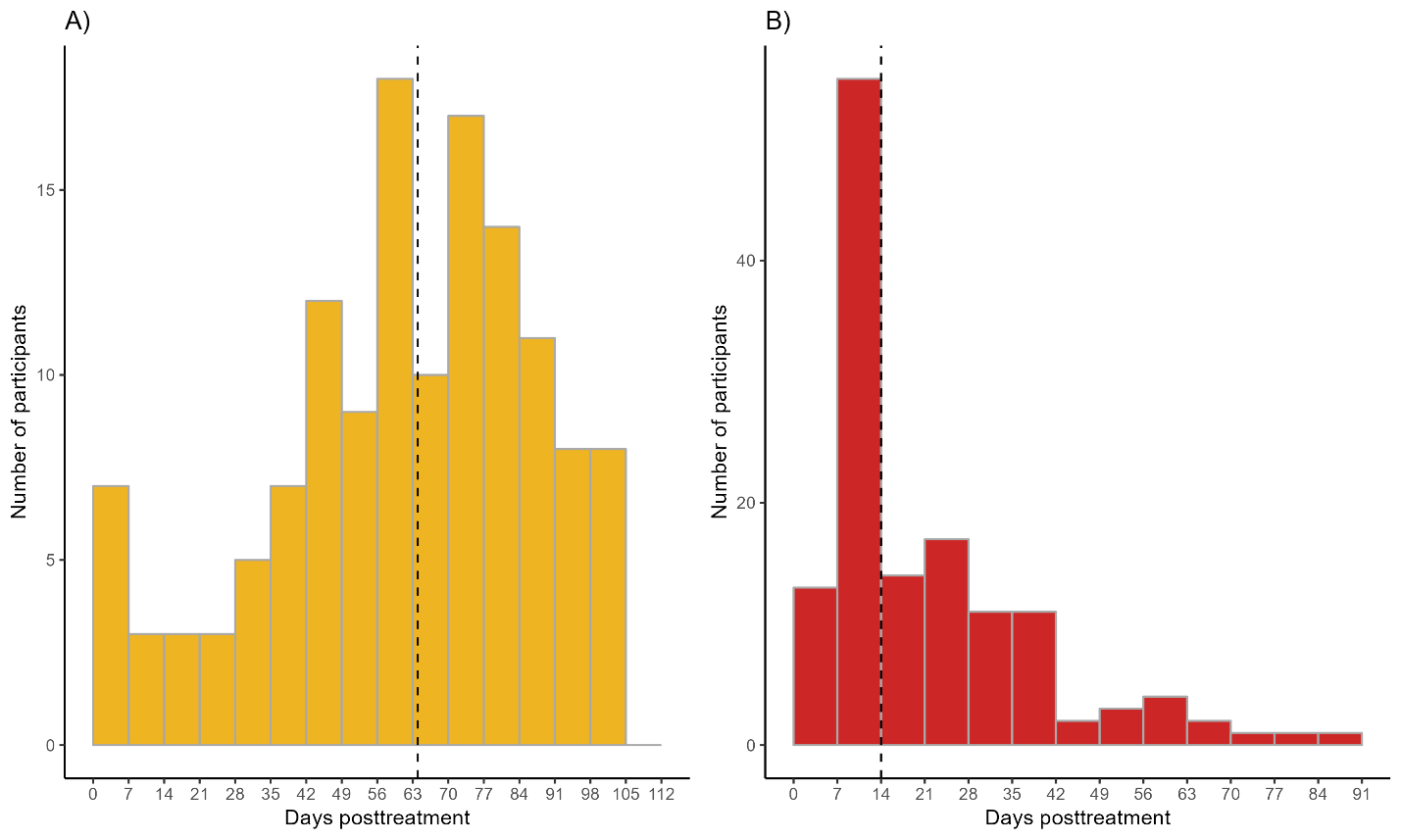

**Supplementary Fig. 3** Histogram of the time post-treatment to reach a threshold of 80 pg/ml for each antigen. HRP2 is shown in panel A on the left, pLDH is shown in panel B on the right. The dashed lines indicate the mean time to reach 80 pg/ml for each antigen.

|  | AIC | BIC |
| --- | --- | --- |
| HRP2 | | |
| Biphasic | 2991.883 | 3017.753 |
| Monophasic | 3068.924 | 3089.62 |
| pLDH | | |
| Biphasic | 2458.063 | 2483.932 |
| Monophasic | 3561.096 | 3581.791 |

**Supplementary Table 1** Table displaying AIC and BIC for the biphasic and monophasic models respectively for each target antigen.

| **Test** | **Antigen concentration at which test has 90% probability of positivity** | | | |
| --- | --- | --- | --- | --- |
|  | **HRP2 (pg/mL)** | | **PfLDH (pg/mL)** | |
|  | **Median estimate** | **95% credible interval** | **Median estimate** | **95% credible interval** |
| Rapigen Pf (pLDH/HRPII) | 525 | (407-661) | 1,318 | (1,175-1,479) |
| Rapigen Pf/Pv (pLDH/pLDH) | - | - | 372 | (302-477) |
| WHO PQ comparator HRP2/PfLDH RDT | 1,072 | (955-1,202) | 5,754 | (5,012-6,607) |

**Supplementary Table 2** Table displaying HRP2 and PfLDH antigen concentrations identified at 90% probability of positivity for Rapigen BIOCREDIT and WHO PQ comparator RDTs. Adapted from Golden et al. [16].
